# Prognostic Role of Pre-Treatment Serum AFP-L3% in Hepatocellular Carcinoma: Systematic Review and Meta-Analysis

**DOI:** 10.1371/journal.pone.0087011

**Published:** 2014-01-30

**Authors:** Jiwen Cheng, Wanli Wang, Yingjun Zhang, Xi Liu, Muxing Li, Zheng Wu, Zhengwen Liu, Yi Lv, Bo Wang

**Affiliations:** 1 Department of Hepatobiliary Surgery, First Affiliated Hospital, School of Medicine, Xi’an Jiaotong University, Xi’an, Shaanxi, China; 2 Department of General Surgery, Bazhong Central Hospital, Bazhong, Sichuan, China; 3 Institute of Advanced Surgical Technology and Engineering, Xi’an Jiaotong University, Xi’an, Shaanxi, China; 4 Department of Infectious Diseases, First Affiliated Hospital, School of Medicine, Xi’an Jiaotong University, Xi’an, Shaanxi, China; 5 Department of Pathology, First Affiliated Hospital, School of Medicine, Xi’an Jiaotong University, Xi’an, Shaanxi, China; Taipei Medical University, Taiwan

## Abstract

**Background:**

Serum lens culinaris agglutinin-reactive fraction of α-fetoprotein (AFP-L3%) has been widely used for HCC diagnosis and follow-up surveillance as tumor serologic marker. However, the prognostic value of high pre-treatment serum AFP-L3% in patients with hepatocellular carcinoma (HCC) remains controversial. We therefore conduct a meta-analysis to assess the relationship between high pre-treatment serum AFP-L3% and clinical outcome of HCC.

**Methods:**

Eligible studies were identified through systematic literature searches. A meta-analysis of fifteen studies (4,465 patients) was carried out to evaluate the association between high pre-treatment serum AFP-L3% and overall survival (OS) and disease-free survival (DFS) in HCC patients. Sensitivity and subgroup analyses were also conducted in this meta-analysis.

**Results:**

Our analysis results showed that high pre-treatment serum AFP-L3% implied poor OS (HR: 1.65, 95%CI: 1.45–1.89 p<0.00001) and DFS (HR: 1.80, 95% CI: 1.49–2.17 p<0.00001) of HCC. Subgroup analysis revealed that there was association between pre-treatment serum AFP-L3% and endpoint (OS and DFS) in low AFP concentration HCC patients (HR: 1.96, 95% CI: 1.24–3.10, p = 0.004; HR: 2.53, 95% CI: 1.09–5.89, p = 0.03, respectively).

**Conclusion:**

The current evidence suggests that high pre-treatment serum AFP-L3% levels indicated a poor prognosis for patients with HCC and AFP-L3% may have significant prognostic value in HCC patients with low AFP concentration.

## Introduction

Hepatocellular carcinoma (HCC) is one of the most frequent neoplasms worldwide, the fifth most prevalent malignancy and the third common cause of cancer-related deaths in the world [1]. Despite of the significant advances in surgical techniques, anesthesia and medical care, better perioperative managements, and new antineoplastic drugs for clinical use, the overall survival of HCC patients remains dismal due to a high rate of recurrence or intrahepatic metastasis after effective treatments [2]. While incidence rates are declining for most cancer sites, HCC is increasing among both men and women [1]. Thus, it is important to identify molecular predictive markers for the prognosis and monitoring metastatic recurrence, which is helpful in the selection of therapeutic strategies and can further improve the survival for HCC patients. Regrettably, there is no widely accepted approach to regular surveillance HCC in the worldwide. Currently, the only generally available serologic marker for HCC surveillance, diagnosis, and monitoring is serum α-fetoprotein (AFP). The combination of ultrasonography (US) and AFP is commonly used for surveillance of HCC. However, it has been recognized that AFP has limited sensitivity and specificity for HCC while US is an indirect diagnostic method depending on operator skill and has limited ability to differentiate HCC from nonneoplastic nodules [3], [4]. Many centers use multidetector CT or dynamic MR imaging and AFP. However, there are no data to support the use of them for surveillance.

Recently, the lens culinaris agglutinin-reactive fraction of α-fetoprotein (AFP-L3) and des-γ-carboxy prothrombin (DCP) have been widely used for HCC diagnosis and follow-up surveillance as tumor serologic markers in Japan. It has been reported that DCP levels have been associated to portal vein invasion and advanced tumoral stage, a fact that prevents the usage of this marker for early detection and prognostic surveillance [5]. Moreover, subsequent studies have shown that positive pre-treatment serum AFP-L3% predicts tumor progression, recurrence and poor clinical outcome [6]–[8], and it has superior prognostic accuracy as a tumor marker compared with AFP or DCP [9], [10]. Nevertheless, conflicting data have emerged regarding the ability of pre-treatment serum AFP-L3% to predict disease-free survival (DFS) and overall survival (OS) in HCC. Therefore, it is necessary to perform a meta-analysis to comprehensively and systematically understand the prognostic value of pre-treatment serum AFP-L3% in HCC.

In this study, we sought to conduct a systematic review and meta-analysis to estimate the prognostic importance of elevated pre-treatment serum AFP-L3 levels for OS and DFS among patients with HCC.

## Materials and Methods

### Literature Search

Studies were identified by electronic searching PubMed, Cochrane Library, EMBASE, and Science Citation Index Expanded databases (last search updated to June 30, 2013). The primary search was based on the random combination of following terms ‘Lens culinaris agglutinin-reactive a-fetoprotein or AFP-L3’, ‘hepatocellular carcinoma or HCC’ and ‘prognosis, survival or recurrence’. For references of identified trials, hand-search was used. Investigators were contacted and asked to supply additional data when key information relevant to the meta-analysis was missing.

### Study Inclusion Criteria

Inclusion criteria were determined by two researchers (JC and WW). Studies eligible for inclusion in this meta-analysis if they met the following criteria: (i) English language; (ii) proven diagnosis of HCC in patients; (iii) provide specific information on survival such as HR/logHR and 95% confidence interval (CI)/standard error (SE) or crude data; (iv) the end-points were DFS or OS; (v) have a maximum follow-up time exceeding 2 years.

### Data Extraction and Quality Assessment

Data were extracted independently by two reviewers (JC and WW) and validated by a third one (YL). Simultaneously, if we found studies that stemmed from overlapping populations, the largest/newest study was included.

The primary goal of this meta-analysis was to detect the prognostic effect of pre-treatment ALP-L3% elevation on OS and DFS among HCC patients. For the studies included in this meta-analysis, the outcome measures were OS or DFS. OS was defined as the interval between the medical treatment (including liver resection, transarterial chemoembolisation, radiofrequency ablation, or liver transplantation, etc.) and the death of patients or the last observation. DFS was designated as the date of treatment until the detection of the recurrence tumor or the last follow-up assessment. To exactly evaluate the value of AFP-L3% in predicting patient outcomes from different treatments, we divided eligible studies into three groups: surgical treatment group in which all HCC patients received curative hepatic resection, and radiofrequency ablation (RFA) treatment group with patients treated with RFA, and multiple treatments group with patients treated by different treatment methods (surgery or RFA or transarterial chemoembolisation (TACE) or percutaneous ethanol injection therapy (PEIT) ).

Quality assessment was conducted in each of the available studies by using the validated Newcastle-Ottawa Quality Assessment Scale for cohort and case–control studies [11] as recommended by the Cochrane Collaboration Guidelines for the assessment of non-randomized studies [12]. The scores range from 0 to 9. Among them, 0 and 9 scores were respectively designated as lowest and highest quality. The studies with 6 scores or more were graded as the high quality ones in the scale [13].

### Data Analysis/synthesis

Review Manager (RevMan) 5.0 (The Cochrane Collaboration, Oxford, England) and Stata version 11 (StataCorp LP, TX) were used in the meta-analysis. All reported p-values were two sided.

The included studies were divided into two groups for analysis: those with data regarding OS and those regarding DFS. Data on the predictive ability of elevated pre-treatment serum AFP-L3% (the cut-off value for elevated serum AFP-L3% was determined by the investigators of each study) for OS and DFS in HCC patients were combined across studies. For the quantitative aggregation of the survival results, hazard ratios (HR) and their 95% confidence intervals (CIs) were combined to give the effective value. If these statistics were not given explicitly in articles, they were calculated from available numerical data using methods reported by Parmar, et al [14]. A Chi-squared test was used to assess the heterogeneity. A *I* ^2^ statistic index greater than 50% and a *x^2^* p-value less than 0.10 indicated the presence of significant heterogeneity [15]. The random-effect model was used to take between-study variation into consideration. Although this does not necessarily rule out the effect of heterogeneity between studies, one may expect a very limited influence. If substantial heterogeneity existed, subgroup analysis was then conducted to explore the potential sources of heterogeneity. Begger’s funnel plot and Egger’s test were performed to assess the publication bias of the eligible studies, where p<0.05 indicated the presence of publication bias [16].

Sensitivity analyses were also conducted by excluding each study individually (the results shown as the summary HR of eligible studies after excluding the one with largest effect and omitting the eligible studies with low quality (NOS score <6) or small sample size (<100). In addition, subgroup analyses were performed to investigate the prognostic impact of pre-treatment serum AFP-L3% alterations on HCC patients in different therapeutic method, HBV/HCV infection, AFP-L3 detection method, AFP concentration and study design.

## Results

### Selection and Characteristics of Studies

**Figure 1:**
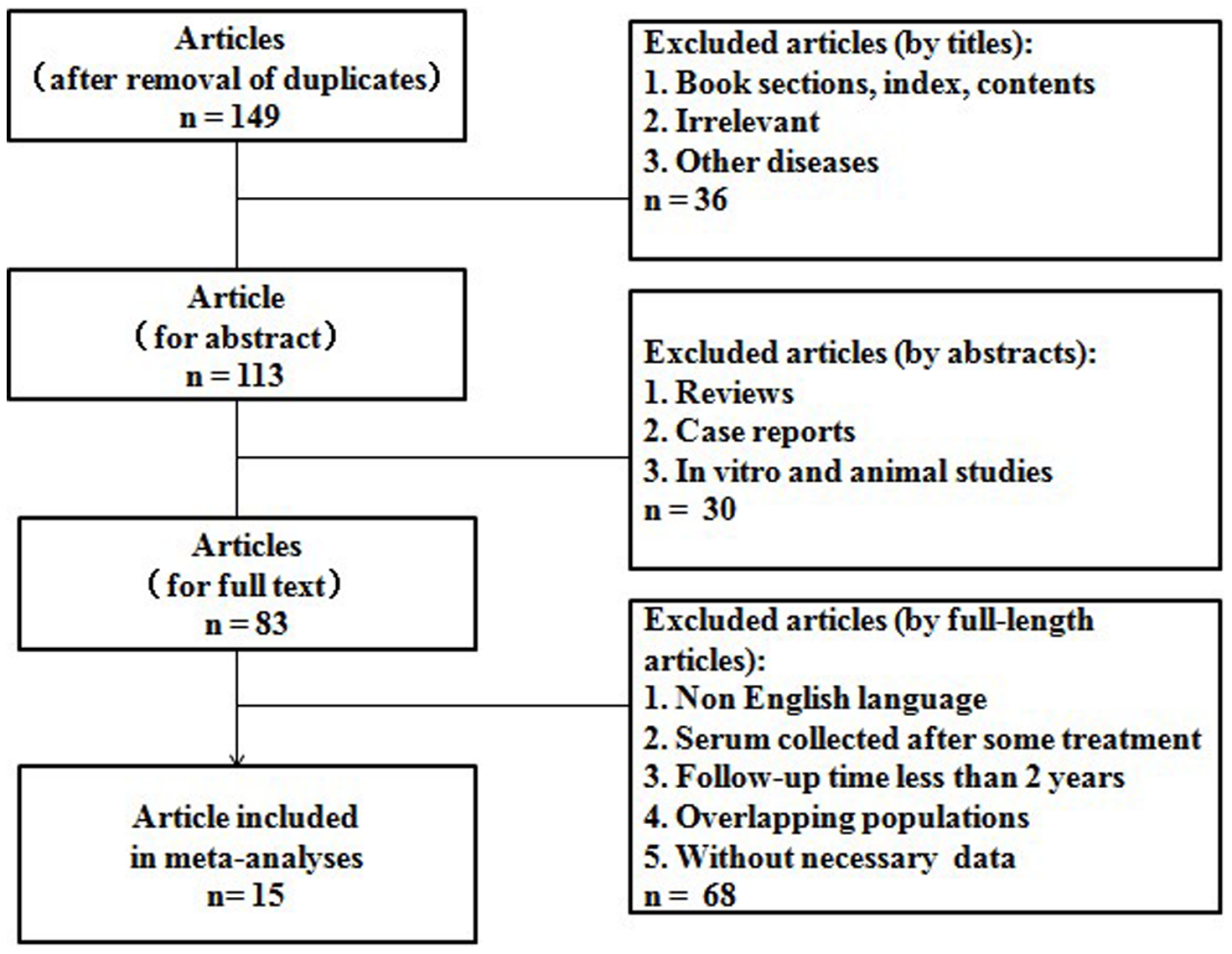
Flow chart showing the inclusion and exclusion process of literatures.

After the careful screening process, 15 studies met our inclusion criteria and were selected for our final meta-analysis [6]–[10], [17]–[26]. The inclusion and exclusion process of the studies is shown in Fig. 1. It is worth pointing out that one of the 15 studies consisted of two subgroups (Surgery group and RFA group) and their data were given respectively, so we analyzed the two subrgroups separately [21]. For the study that only provided the detailed data about RFA on OS and DFS we used only the relevant information in our meta-analysis [19]. Among the 15 studies, one was performed in China [7], one in Korea [6], twelve in Japan [8]–[10], [17]–[24], [26] and one in America [25]; three prospective studies [9], [21], [23], five retrospective studies [8], [17], [18], [24], [25], and seven included studies in which study design was not reported clearly. The basic feature description of the studies was summarized in Table 1.

**Table 1:** Baseline characteristics of the studies in the meta-analysis.

Study(years)	Study design	Treatment	Sample size(M/F,n)	Mean/medianage(years)	Etiology(HCV/HBV)	AFP-L3detection method	Endpoint	Hazard ratios	“High” AFP-L3cut-off level	NOS scores	Factors included in multivariate analysis to identify independent factors influencing survival
Beppu 2010^10^	Unclear	Multiple treatment	108 (77/31)	67.3	89/10	Conventional	DFS	Reported	≥10%	6/9	Alb, AFP-L3, DCP, AST, Plt, PT, ALT
Takeji 2013^17^	R	Multiple treatment	197 (141/56)	65.7	120/42	NR	OS	Reported	≥10%	5/9	NX-PVKA, DCP, NX-PVKA-R, AFP, AFP-L3
Kobayashi 2011^18^	R	Multiple treatment	250 (179/71)	64.0	169/52	Highly sensitive	DFS	Reported	≥5%	7/9	Tumor number, AFP-L3, Albumin, Treatment
Song 2002^6^	Unclear	TACE	46 (39/7)	56.0	6/38	Conventional	OS	Estimated	≥24.4%	6/9	NR
Zhang 2011^7^	Unclear	Surgery	395(281/114)	52.8	31/325	Conventional	DFS/OS	Estimated	≥10%	6/9	NR
Saito 2012^8^	R	Surgery	142(105/37)	64.4	56/50	Conventional	DFS/OS	Reported	≥10%	7/9	AFP, DCP, AFP-L3%, Tumor size, Portal invasion
Nouso 2011^19^	Unclear	RFA*	139	NR	124/16	Highly sensitive	DFS/OS	Estimated	≥10%	6/9	Total bilirubin, Albumin, AST, Plt, Prothrombin time, Ascites, Alcohol, Tumor number, AFP-L3
Toyoda 2012^20^	Unclear	Surgery	173 (136/37)	67.0	116/29	Highly sensitive	DFS/OS	Estimated	≥5%	7/9	Age, Tumor size, Tumor number, Differentiation, Vein invasion, tumor markers (AFP, AFP-L3, and DCP) number
Toyoda 2008^21^	P	Surgery	345 (195/150)	66.0	255/55	Conventional	OS	Reported	≥15%	7/9	Child-Pugh class, Tumor size, Tumor number, AFP-L3, DCP
		RFA	456 (298/158)	68.0	381/34						
Fukuda 1998^22^	Unclear	PEIT	41 (29/12)	63.0	38/3	Conventional	OS	Estimated	≥15%	7/9	Tumor stage, AFP-L3
Shiina 2012^23^	P	RFA	1170 (751/419)	68.3	870/127	NR	OS	Reported	>15%	7/9	Age, Anti-HCV-positive, Child-Pugh class, Tumor size, Tumor number, DCP, AFP-L3, Local tumor progression, Distant recurrence, Plt, AFP
Shiozawa 2009^24^	R	RFA	138(102/36)	68.2	111/13	NR	DFS	Reported	≥10%	6/9	Tumor size, AFP-L3, Tumor location, Ablative margin
Leerapun 2007^25^	R	Multiple treatment	272 (180/92)	NR	58/11	Conventional	OS	Estimated	≥10%	6/9	NR
Tamura 2010^26^	Unclear	Multiple treatment	295 (200/95)	70.0	172/69	Both	OS	Reported	≥7%	6/9	Age, Gender, HBsAg, Anti-HCV, Alcohol intake, Child–Pugh class,AFP, AFP-L3, DCP, tumor size, Tumor number, Vessel invasion, Treatment
Kobayashi 2007^9^	P	Multiple treatment	298 (200/98)	62.0	170/78	Conventional	OS	Reported	≥15%	7/9	DCP, Tumor stage, AFP-L3, Platelet, ALT, Child-Pugh, Indocyanin green disappearance rate, AFP, Age

P, prospective; R, retrospective; M, male; F, female; NR, not reported; DFS, disease-free survival; OS, overall survival; TACE, transarterial chemoembolisation, RFA, radiofrequency ablation; PEIT, percutaneous ethanol injection therapy; AFP, α-fetoprotein; AFP-L3, lens culinaris agglutinin-reactive fraction of α-fetoprotein; HBV, hepatitis B virus; HCV, hepatitis C virus; NOS, The Newcastle-Ottawa Scale Score. Alb, albumin; DCP, des-Á-carboxy prothrombin; ALT, alanine aminotransferase; PT, prothrombin time; Plt, platelet counts; PVKA, protein induced by the absence of vitamin K or antagonist.

### High Pre-treatment Serum AFP-L3% and OS in HCC

**Figure 2:**
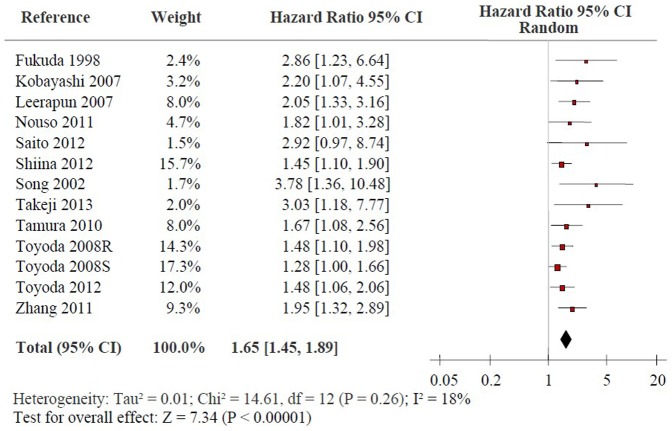
Forrest plot of the association between high pre-treatment serum AFP-L3% and OS of HCC patients. Hazard ratio and associated 95% confidence interval were calculated. OS: overall survival; AFP-L3: the lens culinaris agglutinin-reactive fraction of α-fetoprotein; HCC: hepatocellular carcinoma.

Fig. 2 shows the forest plot for the association between high pre-treatment serum AFP-L3% and OS in HCC. Twelve studies reported the data on high pre-treatment serum AFP-L3% and OS in HCC [6]–[9], [17], [19]–[23], [25], [26]. Combined data showed that high serum AFP-L3% were significantly correlated with OS with a pooled HR estimate of 1.65 [95% confidence interval (CI): 1.45–1.89, p<0.00001] without strong evidence on the presence of heterogeneity (*x^2^* = 14.61, *I* ^2^ = 18%, p = 0.26). Subgroup analysis indicated that elevated pre-treatment serum AFP-L3% levels were significantly associated with OS in HCC patients treated by surgical resection (HR: 1.54, 95% CI: 1.21–1.96, p = 0.0004) while no significant heterogeneity exists (*x^2^* = 4.59, *I* ^2^ = 35%, p = 0.20). Moreover, high pre-treatment serum AFP-L3% exhibited strong association with OS in HCC patients treated by RFA and multiple treatment (HR: 1.50, 95% CI: 1.24–1.81, p<0.0001; HR: 1.97, 95% CI: 1.51–2.58, p<0.00001, respectively) without heterogeneity in the data (*x^2^* = 0.49, *I* ^2^ = 0%, p = 0.78; *x^2^* = 1.52, *I* ^2^ = 0%, p = 0.68, respectively). Sensitivity analyses were performed by considering only the studies with sample size≥100 and NOS score ≥6 and excluding the study with the largest effect size. The summary HRs of the eligible studies was not altered similarly to the overall effect of the meta-analysis (Table 2).

**Table 2:** The results of meta-analyses and sensitivity analyses.

Analysis	No. ofstudies	Pooled hazardratio (95% CI)	*I* ^2^ statistic(%)	*x* ^2^ p-value forheterogeneity	p-value foroverall effect	Analyticalmodel
**Primary analyses**						
*OS*						
AFP-L3% elevation^6–9,17,19–23,25,26^	12	1.65 (1.45–1.89)	18	0.26	p<0.00001	REM
*DFS*						
AFP-L3% elevation^7,8,10,18–20,24^	7	1.80 (1.49–2.17)	0	0.57	p<0.00001	REM
**Sensitivity analyses**						
*AFP-L3% elevation and OS*						
Exclusion of study with the largest effect size^21^	11	1.70 (1.50–1.94)	0	0.45	p<0.00001	REM
Sample size ≥100^7–9,17,19–21,23,25,26^	10	1.57 (1.40–1.77)	0	0.44	p<0.00001	REM
NOS scoring ≥6^6–9,19–23,25,26^	11	1.62 (1.42–1.85)	14	0.30	p<0.00001	REM
*AFP-L3% elevation and DFS*	Not applicable					

CI, confidence interval; REM, random-effect model; DFS, disease-free survival; OS, overall survival; AFP-L3, lens culinaris agglutinin-reactive fraction of α-fetoprotein; NOS, The Newcastle-Ottawa Scale Score.

### High Pre-treatment Serum AFP-L3% and DFS in HCC

**Figure 3:**
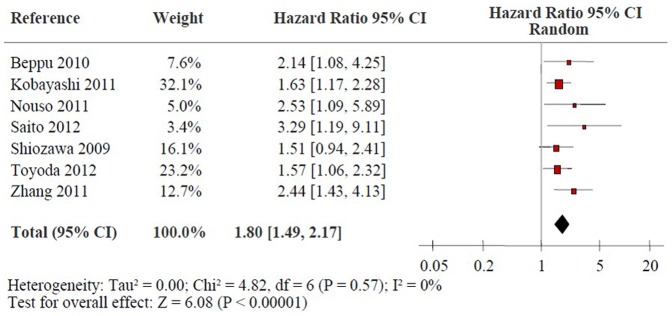
Forrest plot of the association between high pre-treatment serum AFP-L3% and DFS of HCC patients. Hazard ratio and associated 95% confidence interval were calculated. DFS: disease-free survival; AFP-L3: the lens culinaris agglutinin-reactive fraction of α-fetoprotein; HCC: hepatocellular carcinoma.

Fig. 3 shows the forest plot for the association between high pre-treatment serum AFP-L3% and DFS in HCC. Seven of the studies reported data on high pre-treatment serum AFP-L3% and DFS in HCC [7], [8], [10], [18]–[20], [24]. High pre-treatment serum AFP-L3% was significantly associated with poor DFS in all studies except the Shiozawa’s study. The combined data suggested that elevated pre-treatment serum AFP-L3% levels were significantly correlated with DFS with a pooled HR estimate of 1.80 (95% CI: 1.49–2.17, p<0.00001), and thestatistical tests did not support heterogeneity in the data (*x^2^* = 4.82, *I* ^2^ = 0%, p = 0.57). Subgroup analysis indicated that high pre-treatment AFP-L3% levels were significantly related to DFS in HCC treated by surgical resection (HR: 2.02, 95% CI: 1.37–2.99, p = 0.0004), without significant heterogeneity in the data (*x^2^* = 2.87, *I* ^2^ = 30%, p = 0.24). Moreover, high pre-treatment serum AFP-L3% was also highly related with DFS in HCC patients treated by RFA and multiple treatment (HR: 1.73, 95% CI: 1.10–2.71, p = 0.02; HR: 1.72, 95% CI: 1.27–2.32, p = 0.0004, respectively), without strong evidence for heterogeneity (*x^2^* = 1.12, *I* ^2^ = 10%, p = 0.29; *x^2^* = 0.48, *I* ^2^ = 0%, p = 0.49, respectively). Since there is no significant heterogeneity among the eligible articles (*x^2^* = 4.82, *I* ^2^ = 0%, p = 0.57), sensitivity analyses are not applicable.

### Subgroup Analyses

Table 3 shows the detailed results of subgroup analyses. We found that the prognostic effects of high pre-treatment serum AFP-L3% on OS and DFS in HCC patients were similar for different treatment methods, between HBV infection and HCV infection, between highly sensitive and conventional AFP-L3 detection method, and between high AFP concentration and low AFP concentration (*x^2^* p-value for subgroup differences >0.05). However, there was a trend that the summary HR on DFS from the eligible studies on highly sensitive AFP-L3 detection method was distinct from the conventional AFP-L3 detection method, which supported the predictive value of highly sensitive AFP-L3 detection method on DFS (*x^2^* p-value for subgroup differences = 0.08). It is worth noting that the number of eligible studies with data on the correlation of AFP-L3% with low AFP concentration on OS and on DFS is only two and one, respectively. The summary HR of OS and DFS suggested there was association between pre-treatment serum AFP-L3% and endpoint (OS and DFS) in HCC patients with low AFP concentration (HR: 1.96, 95% CI: 1.24–3.10, p = 0.004; HR: 2.53, 95% CI: 1.09–5.89, p = 0.03, respectively). Furthermore, subgroup analysis revealed that the prognostic effects of high pre-treatment serum AFP-L3% on OS and DFS in HCC patients with high AFP concentration and low AFP concentration were similar (*x^2^* p-value for subgroup differences = 0.47, and 0.41, respectively).

**Table 3:** Subgroup analysis of the eligible studies on high pre-treatment serum AFP-L3% associated with OS/DFS in HCC.

Analysis	No. ofstudies	Pooled hazardratio (95% CI)	*I* ^2^ statistic(%)	*x* ^2^ p-value forheterogeneity	*x* ^2^ p-value forsubgroup differences	Analytical model
***OS***						
Therapeutic method						
Surgical resection^7,8,20,21^	4	1.54 (1.21–1.96)	35	0.20		REM
RFA^19,21,23^	3	1.50 (1.24–1.81)	0	0.78		REM
Multiple treatment^9,17,25,26^	4	1.97 (1.51–2.58)	0	0.68	0.24	REM
Viral infection						
HCV infection ≥50%^8,9,17,19–23,25,26^	11	1.57 (1.38–1.79)	7	0.38		REM
HBV infection ≥50%^6,7^	2	2.28 (1.32–3.95)	29	0.24	0.20	REM
AFP-L3 detection method						
Highly sensitive^19,20^	2	1.55 (1.16–2.08)	0	0.54		REM
Conventional^6–9,21,22,25^	8	1.80 (1.44–2.25)	41	0.11	0.43	REM
AFP concentration						
Low^9,19^	2	1.96 (1.24–3.10)	0	0.69		REM
High^6–8,17,20–23,25,26^	11	1.65 (1.42–1.91)	27	0.19	0.47	REM
Study design						
Prospective design^9,21,23^	3	1.42 (1.22–1.66)	0	0.55		REM
Retrospective design^8,17, 25^	3	2.27 (1.57–3.28)	0	0.68	0.02	REM
***DFS***						
Therapeutic method						
Surgical resection^7,8,20^	3	2.02 (1.37–2.99)	30	0.24		REM
RFA^19,24^	2	1.73 (1.10–2.71)	10	0.29		REM
Multiple treatment^10,18^	2	1.72 (1.27–2.32)	0	0.49	0.79	REM
Viral infection						
HBV infection ≥50%^7^	1	2.44 (1.43–4.13)	Not applicable	Not applicable		REM
HCV infection ≥50%^8,10,18–20,24^	6	1.72 (1.40–2.10)	0	0.64	0.23	REM
AFP-L3 detection method						
Highly sensitive^18–20^	3	1.63 (1.32–2.03)	0	0.75		REM
Conventional^7,8,10^	3	2.44 (1.66–3.59)	0	0.79	0.08	REM
AFP concentration						
Low^19^	1	2.53 (1.09–5.89)	Not applicable	Not applicable		REM
High^7,8,10,18,20,24^	6	1.76 (1.45–2.14)	0	0.53	0.41	REM
Study design						
Retrospective design^8,18, 24^	3	1.67 (1.28–2.17)	0	0.39		REM

CI, confidence interval; REM, random-effect model; DFS, disease-free survival; OS, overall survival; RFA, radiofrequency ablation; AFP, α-fetoprotein; AFP-L3, lens culinaris agglutinin-reactive fraction of α-fetoprotein; HBV, hepatitis B virus; HCV, hepatitis C virus.

### Publication Bias

Publication bias estimate was mainly used to evaluate the reliability of meta-analysis results, especially those showed statistical significance. The assessment of publication bias using Begger’s test provided evidence for publication bias in OS(p = 0.001),but not in DFS studies (p = 0.23) (Fig. S1A and S1B), Egger’s test was used to provide further statistical evidence; however, the results showed obvious publication bias both in OS and DFS studies (p<0.001 and p = 0.022, respectively).

## Discussion

Tumor invasiveness, metastasis and recurrence often result in poor clinical outcome of HCC patients [27]. Thus, it is important to identify molecular predictive markers for prognosis and for monitoring metastatic recurrence, which is helpful in selecting therapeutic strategies and can also improve survival of HCC patients. However, there are no widely accepted serologic markers associated with tumor progression, invasion and recurrence of HCC that are useful for the regular surveillance HCC with regard to treatment and outcomes. One candidate marker for the progression and prognosis of HCC is AFP-L3 [6], [28]. Although the association of pre-treatment AFP-L3 with tumor development and progression has been explored for several years, yet the available data have not been analyzed comprehensively till now. Considered as a valuable tool in biomarker validation, a meta-analysis was carried out to study the predictive value of high pre-treatment serum AFP-L3% on the prognosis of HCC patients.

In this meta-analysis, we firstly evaluated the association of elevated pre-treatment serum AFP-L3% levels with OS and DFS in HCC. Many reports have shown that AFP-L3 status is an independent prognostic factor in patients with HCC [29]–, the elevation of it indicated poor prognosis and decreased survival rates. The results of eight studies[8]–[10], [18], [19], [22], [26] included in this meta-analysis shown that elevated preoperative AFP-L3 was an independent predictor of OS or DFS for HCC, while another four included studies [17], [21], [24], [25] reported the opposite results. The pooled results demonstrated that high pre-treatment serum AFP-L3 levels significantly predicted poor OS and DFS for HCC (p<0.00001, p<0.00001, respectively), which may serve as an independent prognostic factor in patients with HCC. Although the analysis results were positive, the reasons for the differences of OS and DFS among HCC patients with high and low serum AFP-L3 levels were still unclear. It is worth noting that many factors were usually correlated with poor prognosis of HCC, such as tumor invasion, metastasis, extent of cirrhosis, and treatment methods. Unfortunately, the relation between pre-treatment serum AFP-L3% levels and tumor invasion, metastasis and other clinicopathological parameters (such as tumor grade, stage, and cirrhosis) reported in those studies were not reliable, so we failed to estimate the association between pre-treatment serum AFP-L3% and those factors and can not determine the influence of these factors related to AFP-L3 level and OS or DFS. In this study, the subgroup analyses showed that high serum AFP-L3% levels was also significantly related with OS and DFS in HCC treated by surgical resection (p = 0.0004, p = 0.0004, respectively), RFA (p<0.0001, p = 0.02, respectively), or multiple treatment (p<0.00001, p = 0.0004, respectively). Furthermore, AFP-L3% levels were not found significantly different for surgically and non-surgically treated groups. Intriguingly, AFP-L3% was found to be a useful prognostic marker in AFP negative HCC [9], [19]. We also noticed that the AFP-L3% level obtained by conventional measurement methods is unstable or even undetectable in cases with low AFP concentration. Most recently, a novel automated immunoassay for AFP-L3 has been developed [34], [35], which enabled us to overcome this difficulty and showed that AFP-L3% was closely related to poor prognosis even in AFP negative HCC. In this meta-analysis, there are only two studies and one study respectively providing relevant data on the correlation of AFP-L3 with low AFP concentration on OS and DFS. The summary HRs of OS and DFS suggested that high pre-treatment serum AFP-L3% levels indicated a poor prognosis for patients with AFP negative HCC. Although the subgroup analysis revealed that the prognostic effects of high serum AFP-L3% on OS and DFS in HCC patients were similar for highly sensitive and conventional AFP-L3 detection method (*x^2^* p-value for subgroup differences >0.05), there was a trend that the summary HR on DFS from eligible studies on highly sensitive AFP-L3 detection method was distinct from the conventional AFP-L3 detection method, supporting the predictive value of highly sensitive AFP-L3 detection method on DFS (*x^2^* p-value for subgroup differences = 0.08). Thus, whether the highly sensitive AFP-L3 detection method were superior to conventional AFP-L3 detection method in predicting HCC prognosis remained to be investigated by further studies.

Recently, des-γ-carboxy prothrombin (DCP),one of tumor markers specific to HCC, has also been widely used to predict early postoperative recurrence and poor prognosis [21], [36], [37]. Ten of studies included in this meta-analysis also assessed the prognostic significance of DCP in HCC. Thus, we combined the data of DCP and compared its prognostic value with AFP-L3 for HCC. The results in this meta-analysis suggest that serum AFP-L3% may have better prognostic value than serum DCP for the HCC (data not shown). However, whether the combination of AFP-L3 with other markers will have an improved predictive ability for estimating survival in HCC remained to be assessed by further studies.

Although we assessed comprehensively the prognostic significance of pre-treatment AFP-L3% in HCC, some limitations in our meta-analysis should be discussed. Firstly, potential language and risk bias may exist in this systematic review, because positive study results were more often published than negative ones and we only sought reports written in English. In addition, although we tried to identify all relevant information, some missing data were still unavoidable. Furthermore, our publication bias estimate using both Begger’s plot and Egger’s test showed that publication bias exists in studies on high serum AFP-L3 levels associated with OS and RFS. Secondly, aetiological bias was a concern, because most of the cohorts of eligible studies were hepatitis C virus-related HCC. Hence, we cannot know if AFP-L3 has the same performance in hepatitis B virus-related HCC. Thirdly, five retrospective studies [8], [17], [18], [24], [25] included in this meta-analysis. Although the results of both prospective and retrospective design subgroup analysis shown that high pre-treatment serum AFP-L3% implied poor OS and DFS without significant heterogeneity in the data (p-value for overall effect <0.05), validation of the prognostic value of AFP-L3% in HCC in more prospective studies is required. In addition, liver cirrhosis, tumour stage and Portal Vein Tumor Thrombus (PVTT) were considered to be related to clinical outcomes in HCC patients. Unfortunately, the relation of high pre-treatment serum AFP-L3% and these important prognostic factors in HCC were not evaluated in this meta-analysis due to incomplete data. Finally, although the results of this meta-analysis suggested that pre-treatment AFP-L3% may have significant prognostic value in HCC patients with low AFP concentration, the external validity of results and applicability requires more studies to be done, because only two studies assessed the prognostic value of AFP-L3% in HCC with low AFP concentration in our meta-analysis. Therefore, we suggest that our results should be interpreted cautiously.

In conclusion, our meta-analysis suggested that high pre-treatment serum AFP-L3% levels indicated a poor prognosis for patients with HCC, and pre-treatment AFP-L3 may have significant prognostic value in HCC patients with low AFP concentration.

## Supporting Information

Figure S1 A **Bias assessment plots for studies (OS) included in our meta-analysis. B. Bias assessment plots for studies (DFS) included in our meta-analysis.**
(TIF)

Checklist S1 **PRISMA Checklist.**
(PDF)

## References

[1] Siegel R, Naishadham D, Jemal A (2013) Cancer statistics, 2013. CA Cancer J Clin 63: 11–30. 23335087 10.3322/caac.21166

[2] Tang ZY, Ye SL, Liu YK, Qin LX, Sun HC, et al (2004) A decade’s studies on metastasis of hepatocellular carcinoma. J Cancer Res Clin Oncol 130: 187–196. 14685850 10.1007/s00432-003-0511-1 PMC12161827

[3] Bruix J, Sherman M, Llovet JM, Beaugrand M, Lencioni R, et al (2001) Clinical management of hepatocellular carcinoma. Conclusions of the Barcelona-2000 EASL conference. European Association for the Study of the Liver. J Hepatol 35: 421–430. 11592607 10.1016/s0168-8278(01)00130-1

[4] Forner A, Llovet JM, Bruix J (2012) Hepatocellular carcinoma. Lancet 379: 1245–1255. 22353262 10.1016/S0140-6736(11)61347-0 PMC13537732

[5] Koike Y, Shiratori Y, Sato S, Obi S, Teratani T, et al (2001) Des-gamma-carboxy prothrombin as a useful predisposing factor for the development of portal venous invasion in patients with hepatocellular carcinoma: a prospective analysis of 227 patients. Cancer 91: 561–569. 11169939 10.1002/1097-0142(20010201)91:3<561::aid-cncr1035>3.0.co;2-n

[6] Song BC, Suh DJ, Yang SH, Lee HC, Chung YH, et al (2002) Lens culinaris agglutinin-reactive alpha-fetoprotein as a prognostic marker in patients with hepatocellular carcinoma undergoing transcatheter arterial chemoembolization. J Clin Gastroenterol 35: 398–402. 12394228 10.1097/00004836-200211000-00008

[7] Zhang XF, Lai EC, Kang XY, Qian HH, Zhou YM, et al (2011) Lens culinaris agglutinin-reactive fraction of alpha-fetoprotein as a marker of prognosis and a monitor of recurrence of hepatocellular carcinoma after curative liver resection. Ann Surg Oncol 18: 2218–2223. 21336512 10.1245/s10434-011-1613-7

[8] Saito Y, Shimada M, Utsunomiya T, Morine Y, Imura S, et al (2012) Prediction of recurrence of hepatocellular carcinoma after curative hepatectomy using preoperative Lens culinaris agglutinin-reactive fraction of alpha-fetoprotein. Hepatol Res 42: 887–894. 22524419 10.1111/j.1872-034X.2012.01004.x

[9] Kobayashi M, Kuroiwa T, Suda T, Tamura Y, Kawai H, et al (2007) Fucosylated fraction of alpha-fetoprotein, L3, as a useful prognostic factor in patients with hepatocellular carcinoma with special reference to low concentrations of serum alpha-fetoprotein. Hepatol Res 37: 914–922. 17610501 10.1111/j.1872-034X.2007.00147.x

[10] Beppu T, Sugimoto K, Shiraki K, Tameda M, Kusagawa S, et al (2010) Clinical significance of tumor markers in detection of recurrent hepatocellular carcinoma after radiofrequency ablation. Int J Mol Med 26: 425–433. 20664960

[11] Wells GA, Shea B, O’Connell D, Peterson J, Welch V, et al.(1999) The Newcastle-Ottawa Scale (NOS) for assessing the quality of nonrandomised studies in meta-analyses. Available: http://www.ohri.ca/programs/clinical_epidemiology/oxford_web.ppt. Accessed 26 May 2013.

[12] Reeves BC, Higgins J, Ramsay C, Shea B, Tugwell P, et al (2013) An introduction to methodological issues when including non-randomised studies in systematic reviews on the effects of interventions. Res Syn Meth 4: 1–11. 10.1002/jrsm.1068 26053535

[13] Liu J, Ma Q, Zhang M, Wang X, Zhang D, et al (2012) Alterations of TP53 are associated with a poor outcome for patients with hepatocellular carcinoma: evidence from a systematic review and meta-analysis. Eur J Cancer 48: 2328–2338. 22459764 10.1016/j.ejca.2012.03.001 PMC3395767

[14] Parmar MK, Torri V, Stewart L (1998) Extracting summary statistics to perform meta-analyses of the published literature for survival endpoints. Stat Med 17: 2815–2834. 9921604 10.1002/(sici)1097-0258(19981230)17:24<2815::aid-sim110>3.0.co;2-8

[15] Thompson SG, Higgins JP (2002) How should meta-regression analyses be undertaken and interpreted? Stat Med 21: 1559–1573. 12111920 10.1002/sim.1187

[16] Egger M, Davey Smith G, Schneider M, Minder C (1997) Bias in meta-analysis detected by a simple, graphical test. BMJ 315: 629–634. 9310563 10.1136/bmj.315.7109.629 PMC2127453

[17] Takeji S, Hirooka M, Koizumi Y, Tokumoto Y, Abe M, et al (2013) Des-gamma-carboxy prothrombin identified by P-11 and P-16 antibodies reflects prognosis for patients with hepatocellular carcinoma. J Gastroenterol Hepatol 28: 671–677. 23215762 10.1111/jgh.12076

[18] Kobayashi M, Hosaka T, Ikeda K, Seko Y, Kawamura Y, et al (2011) Highly sensitive AFP-L3% assay is useful for predicting recurrence of hepatocellular carcinoma after curative treatment pre- and postoperatively. Hepatol Res 41: 1036–1045. 21883741 10.1111/j.1872-034X.2011.00858.x

[19] Nouso K, Kobayashi Y, Nakamura S, Kobayashi S, Takayama H, et al (2011) Prognostic importance of fucosylated alpha-fetoprotein in hepatocellular carcinoma patients with low alpha-fetoprotein. J Gastroenterol Hepatol 26: 1195–1200. 21410750 10.1111/j.1440-1746.2011.06720.x

[20] Toyoda H, Kumada T, Tada T, Niinomi T, Ito T, et al (2012) Prognostic significance of a combination of pre- and post-treatment tumor markers for hepatocellular carcinoma curatively treated with hepatectomy. J Hepatol 57: 1251–1257. 22824818 10.1016/j.jhep.2012.07.018

[21] Toyoda H, Kumada T, Kaneoka Y, Osaki Y, Kimura T, et al (2008) Prognostic value of pretreatment levels of tumor markers for hepatocellular carcinoma on survival after curative treatment of patients with HCC. J Hepatol 49: 223–232. 18571271 10.1016/j.jhep.2008.04.013

[22] Fukuda H (1998) Tumor vascularity and lens culinaris agglutinin reactive alpha-fetoprotein are predictors of long-term prognosis in patients with hepatocellular carcinoma after percutaneous ethanol injection therapy. Kurume Med J 45: 187–193. 9715046 10.2739/kurumemedj.45.187

[23] Shiina S, Tateishi R, Arano T, Uchino K, Enooku K, et al.. (2012) Radiofrequency ablation for hepatocellular carcinoma: 10-year outcome and prognostic factors. Am J Gastroenterol 107: 569–577; quiz 578. 10.1038/ajg.2011.425 PMC3321437 22158026

[24] Shiozawa K, Watanabe M, Wakui N, Ikehara T, Iida K, et al (2009) Risk factors for the local recurrence of hepatocellular carcinoma after single-session percutaneous radiofrequency ablation with a single electrode insertion. Mol Med Rep 2: 89–95. 21475796 10.3892/mmr_00000067

[25] Leerapun A, Suravarapu SV, Bida JP, Clark RJ, Sanders EL, et al.. (2007) The utility of Lens culinaris agglutinin-reactive alpha-fetoprotein in the diagnosis of hepatocellular carcinoma: evaluation in a United States referral population. Clin Gastroenterol Hepatol 5: 394–402; quiz 267. 10.1016/j.cgh.2006.12.005 PMC1931510 17368240

[26] Tamura Y, Igarashi M, Kawai H, Suda T, Satomura S, et al (2010) Clinical advantage of highly sensitive on-chip immunoassay for fucosylated fraction of alpha-fetoprotein in patients with hepatocellular carcinoma. Dig Dis Sci 55: 3576–3583. 20407827 10.1007/s10620-010-1222-5

[27] Tung-Ping Poon R, Fan ST, Wong J (2000) Risk factors, prevention, and management of postoperative recurrence after resection of hepatocellular carcinoma. Ann Surg 232: 10–24. 10862190 10.1097/00000658-200007000-00003 PMC1421103

[28] Yamashita F, Tanaka M, Satomura S, Tanikawa K (1996) Prognostic significance of Lens culinaris agglutinin A-reactive alpha-fetoprotein in small hepatocellular carcinomas. Gastroenterology 111: 996–1001. 8831594 10.1016/s0016-5085(96)70067-7

[29] Aoyagi Y, Isokawa O, Suda T, Watanabe M, Suzuki Y, et al (1998) The fucosylation index of alpha-fetoprotein as a possible prognostic indicator for patients with hepatocellular carcinoma. Cancer 83: 2076–2082. 9827711

[30] Hayashi K, Kumada T, Nakano S, Takeda I, Sugiyama K, et al (1999) Usefulness of measurement of Lens culinaris agglutinin-reactive fraction of alpha-fetoprotein as a marker of prognosis and recurrence of small hepatocellular carcinoma. Am J Gastroenterol 94: 3028–3033. 10520864 10.1111/j.1572-0241.1999.01378.x

[31] Oka H, Saito A, Ito K, Kumada T, Satomura S, et al (2001) Multicenter prospective analysis of newly diagnosed hepatocellular carcinoma with respect to the percentage of Lens culinaris agglutinin-reactive alpha-fetoprotein. J Gastroenterol Hepatol 16: 1378–1383. 11851836 10.1046/j.1440-1746.2001.02643.x

[32] Tateishi R, Shiina S, Yoshida H, Teratani T, Obi S, et al (2006) Prediction of recurrence of hepatocellular carcinoma after curative ablation using three tumor markers. Hepatology 44: 1518–1527. 17133456 10.1002/hep.21408

[33] Miyaaki H, Nakashima O, Kurogi M, Eguchi K, Kojiro M (2007) Lens culinaris agglutinin-reactive alpha-fetoprotein and protein induced by vitamin K absence II are potential indicators of a poor prognosis: a histopathological study of surgically resected hepatocellular carcinoma. J Gastroenterol 42: 962–968. 18085353 10.1007/s00535-007-2117-x

[34] Kawabata T, Wada HG, Watanabe M, Satomura S (2008) Electrokinetic analyte transport assay for alpha-fetoprotein immunoassay integrates mixing, reaction and separation on-chip. Electrophoresis 29: 1399–1406. 18384019 10.1002/elps.200700898

[35] Kagebayashi C, Yamaguchi I, Akinaga A, Kitano H, Yokoyama K, et al (2009) Automated immunoassay system for AFP-L3% using on-chip electrokinetic reaction and separation by affinity electrophoresis. Anal Biochem 388: 306–311. 19250915 10.1016/j.ab.2009.02.030

[36] Fujiyama S, Tanaka M, Maeda S, Ashihara H, Hirata R, et al (2002) Tumor markers in early diagnosis, follow-up and management of patients with hepatocellular carcinoma. Oncology 62 Suppl 157–63. 11868787 10.1159/000048277

[37] Toyoda H, Kumada T, Kiriyama S, Sone Y, Tanikawa M, et al (2006) Prognostic significance of simultaneous measurement of three tumor markers in patients with hepatocellular carcinoma. Clin Gastroenterol Hepatol 4: 111–117. 16431313 10.1016/s1542-3565(05)00855-4

