# Supplementary figures and images for: Prognostic Role of Pre-Treatment Serum AFP-L3% in Hepatocellular Carcinoma: Systematic Review and Meta-Analysis
Source: PLoS One. 2014 Jan 30;9(1):e87011. doi: 10.1371/journal.pone.0087011 (PMC3907387; doi:10.1371/journal.pone.0087011)

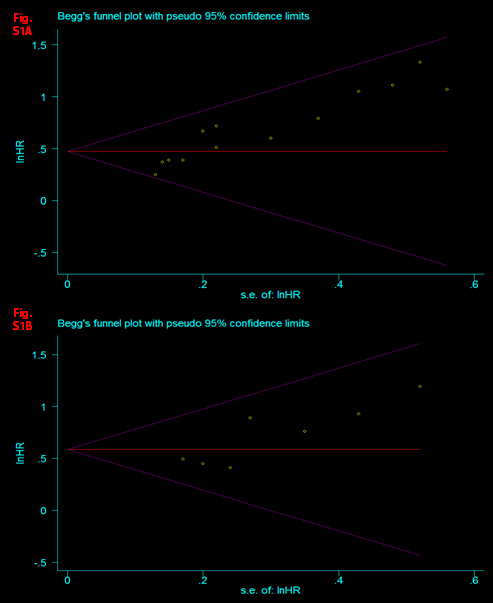

Supplement: Figure S1 A — Bias assessment plots for studies (OS) included in our meta-analysis. B. Bias assessment plots for studies (DFS) included in our meta-analysis. (TIF) [file pone.0087011.s001.tif]
